# Type 1 and type 2 NSTEMI in atrial fibrillation: insights from the HERA-FIB registry

**DOI:** 10.1007/s00392-026-02912-8

**Published:** 2026-04-15

**Authors:** Mustafa Yildirim, Christian Salbach, Johannes Dürr, Matthias Mueller-Hennessen, Hugo A. Katus, Norbert Frey, Evangelos Giannitsis

**Affiliations:** 1 Department of Internal Medicine III, Cardiology, University Hospital of Heidelberg, Heidelberg, 69120 Germany; 2 DZHK (German Centre for Cardiovascular Research), Standort Heidelberg/Mannheim, Heidelberg, 69120 Germany

**Keywords:** Real-world evidence, Atrial fibrillation, NSTEMI, Revascularization, High-sensitivity troponin, Coronary angiography

## Abstract

**Background:**

Differentiating type 1 from type 2 non–ST-elevation myocardial infarction (NSTEMI) is clinically challenging, particularly in patients with atrial fibrillation (AF), where the 4th Universal Definition of Myocardial Infarction (UDMI) allows classification based on presumed mechanisms without mandatory coronary angiography. Real-world data from AF cohorts undergoing systematic invasive evaluation remain limited.

**Methods:**

Between 2009 and 2020, we analyzed consecutive AF patients admitted with NSTEMI to a tertiary care center. Patients undergoing early coronary angiography were included. Infarct subtypes were adjudicated according to the 4th UDMI incorporating angiographic findings. The primary objective was to determine the prevalence of type 1 and type 2 NSTEMI. The secondary endpoint was a composite of all-cause mortality, myocardial infarction, stroke, or major bleeding.

**Results:**

Among 536 eligible patients, 438 (81.7%) underwent coronary angiography. Of these, 312 (71.2%) were classified as type 1 NSTEMI, 100 (22.8%) as type 2 NSTEMI, and 26 (5.9%) as acute myocardial injury. Type 2 NSTEMI was associated with preserved left ventricular function and less extensive coronary artery disease. Over a median follow-up of 2.21 years, composite event rates were 40.1% in type 1 and 36.0% in type 2 NSTEMI. Acute myocardial injury was associated with the highest composite event rate (65.4%) and all-cause mortality (57.7%).

**Conclusions:**

In symptomatic AF patients, type 1 NSTEMI predominates and may be underdiagnosed without angiography, which enables accurate classification and timely revascularization. Type 1 and type 2 NSTEMI confer similarly high long-term risk, while acute myocardial injury identifies a particularly high-risk subgroup requiring systematic evaluation and targeted management.

**Trial registration:**

Heidelberg Registry of Atrial Fibrillation [HERA‑FIB]; NCT05995561.

**Graphical Abstract:**

**Figure:**
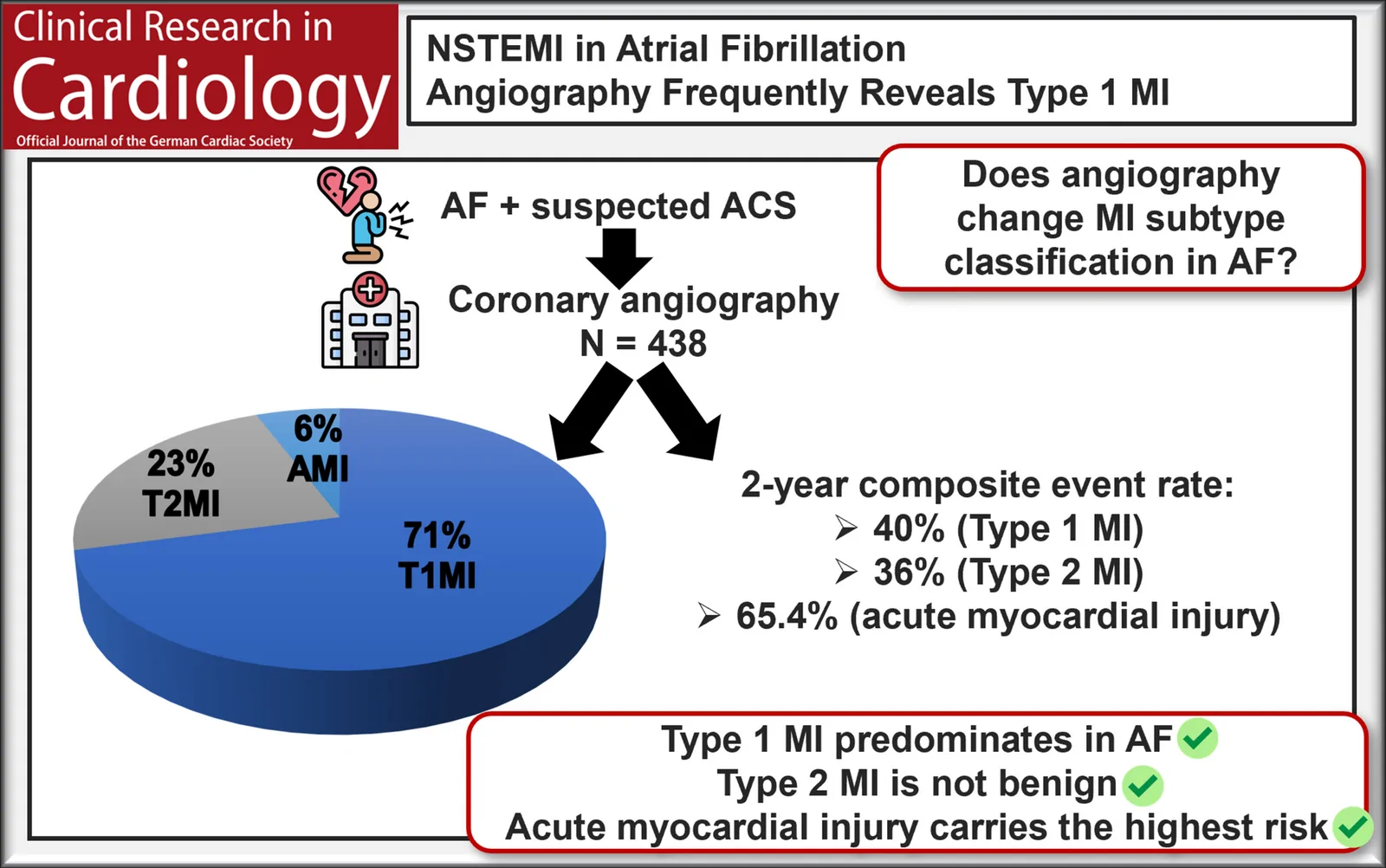

**Supplementary Information:**

The online version contains supplementary material available at https://doi.org/10.1007/s00392-026-02912-8.

## Introduction

The 4th Universal Definition of Myocardial Infarction (UDMI) defines 5 subtypes of myocardial infarction (MI) [1]. Differentiation between type 1 and type 2 MI is most challenging because type 1 MI is characterized by plaque rupture, erosion, dissection or fissure of a coronary plaque and results in an occlusive or subocclusive intracoronary thrombus formation that requires early coronary intervention. In contrast, type 2 MI arises from a mismatch between myocardial oxygen demand and supply, a condition that does not necessarily require the presence of a relevant coronary stenosis [1]. To enable a global adoption of a universal definition of myocardial infarction, the diagnosis of type 2 MI does not require coronary angiography but is assumed by the putative underlying mechanism. As such, symptomatic patients with AF who fulfill the criteria of the 4th UDMI definition, i.e., elevated high-sensitivity cardiac troponin (hs-cTn) above 99th ULN (Upper Limit of Normal) and a relevant rise and/or fall are diagnosed as type 2 MI [2–4]. Consequently, AF patients are frequently categorized as type 2 MI, although the underlying coronary anatomy is often not systematically evaluated [5, 6].

To date, most information on the prevalence, clinical characteristics, and outcomes of patients with type 2 MI comes from studies without or with very low rates of coronary angiography—typically between 10 and 36%—restricting accurate assessment of infarct mechanism and underlying coronary anatomy [5, 7–9]. In particular, type 1 MI may be under-recognized in cohorts where classification is based primarily on clinical judgment or troponin dynamics without systematic angiographic evaluation.

To overcome these limitations, we leveraged a single-center tertiary care registry with a systematic invasive strategy, where 81.7% of AF patients with Non-ST-Elevation Myocardial Infarction (NSTEMI) underwent coronary angiography. This approach provides a unique opportunity to assess the angiographic phenotype of MI subtypes in AF patients presenting with symptoms suggestive of acute coronary syndrome.

Therefore, the primary goal of this study was to assess the prevalence of type 1 and type 2 MI among patients with atrial fibrillation (AF) and NSTEMI where the subtype assignment is based on coronary angiography (CA) at index presentation and application of the angiographic criteria of the 4th UDMI. As secondary endpoints, we analyzed clinical characteristics and a composite of clinical outcomes consisting of all-cause mortality, myocardial infarction, stroke, or major bleeding.

## Methods

### Patients and definitions

The present study is a retrospective analysis of the Heidelberg Registry of Atrial Fibrillation (HERA‑FIB), a single‑center, all‑comer database of consecutive patients presenting with AF at the Heidelberg University Hospital. The HERA‑FIB registry was established to capture demographic, clinical, and procedural data on AF patients and has been described previously [10].

AF was present at the time of index presentation in all cases, accompanied by symptoms suggestive of acute coronary syndrome (ACS).

For this substudy, adults (≥ 18 years) presenting with a broad range of symptoms suggestive of ACS, encompassing palpitations, chest pain or chest discomfort, dyspnea, unexplained diaphoresis, epigastric pain, or pain radiating to the back, arms, or shoulder were enrolled. All patients had documented AF on at least one 12-lead ECG (electrocardiogram) at admission. Patients qualified for inclusion if they underwent coronary angiography within index hospitalization. Patients without at least one high-sensitivity cardiac troponin T (hs-cTnT) measurement, those with stage 5 chronic kidney disease (CKD) with or without hemodialysis, and those who were lost to follow-up were excluded. Repeat admissions were excluded, and participants with missing data for a given analysis were censored from that analysis only.

Patients were not excluded based on angiographic findings; rather, only those not undergoing CA during index hospitalization were excluded from the present analysis, as infarct subtype adjudication required angiographic data.

At our institution, early invasive evaluation is standard for all patients meeting criteria for NSTEMI, unless contraindicated due to advanced comorbidities such as severe cognitive impairment, malignancy with limited prognosis, septic shock, or upon patient refusal. This policy results in a consistently high rate of coronary angiography and minimizes selection bias.

The study was approved by the local ethics committee of the University of Heidelberg. Given the retrospective design of the study, informed consent was waived by the local ethics committee. The study was conducted according to ethical principles stated in the Declaration of Helsinki (2008). Patient identifiable data were pseudonymized to ensure data confidentiality and were not passed on to third parties. This study is registered at ClinicalTrials.gov. ClinicalTrials.gov identifier: NCT05995561.

### Biomarker sampling

By default, all patients presenting to the emergency department (ED) received cardiac troponin testing per European Society of Cardiology (ESC) 0/1-, ESC 0/2-, or ESC 0/3-h protocol as recommended by respective ESC Guidelines throughout the entire study recruitment period between 2009 and 2020 [11, 12]. Hs‑cTnT was measured using hs-cTnT (Roche Diagnostics, Penzberg, Germany) on a COBAS 411 or a COBAS E601 analyser. The assays’ limits of detection (LoD) were 5 ng/L, with a 10% coefficient of variation at 13 ng/L and a 99th percentile upper reference limit of 14 ng/L [13, 14]. Sex‑specific cut‑offs were not applied. Troponin was reported to one decimal. Physicians and medical staff received regular training on all fast ESC algorithms. Timing of serial sampling and adherence to protocol were not systematically monitored.

### Diagnosis of NSTEMI type and chronic myocardial injury

The UDMI criteria were applied for diagnosis of NSTEMI which required an elevation of hs-cTnT above the 99th percentile ULN together with a relevant rise and or fall of cardiac troponin (cTn), together with either symptoms suggestive of an acute myocardial ischemia, ECG changes, or other imaging information suggesting a new ischemic event [1]. The diagnosis of NSTEMI was based exclusively on the third or fourth UDMI, according to the respective guideline version applicable during the recruitment period. ESC 0/1h [11], ESC 0/2h [11], and ESC 0/3h [12] hs-cTnT algorithms were used in routine clinical care to guide early triage and risk stratification, but did not replace UDMI-based diagnostic adjudication.

Patients allocated to the observational zone according to ESC algorithms underwent further clinical assessment and serial hs-cTnT measurements. Only those fulfilling UDMI criteria for myocardial infarction were included in the present analysis.

Patients with other confirmed causes for troponin elevation (“rule-in”) including myocarditis, pulmonary embolism, stress cardiomyopathy were not counted as NSTEMI but were included in the analysis after confirmation of the underlying reason for “rule-in,” regardless of whether they had received an invasive coronary angiography.

In clinical routine, NSTEMI was coded per ICD 10 as MI without differentiation of infarct subtype. Infarct subtypes were adjudicated for the present study at later time points, and all patients received revascularization procedures and pharmacological therapies at the discretion of the responsible physician at the peripheral ward who was not aware of adjudicated infarct subtypes at the time of index hospitalization.

### ESC rapid rule-in and rule-out algorithms

The ESC algorithms are described to reflect routine clinical workflow during the study period. We applied the ESC 0/1h [11], ESC 0/2h [11], and ESC 0/3h [12] hs-cTnT protocols to classify patients with AF presenting with suspected ACS as rule-in or rule-out; all others were allocated to the observational zone. Under the 0/1-h protocol [11], patients were ruled in if the baseline hs-cTnT was ≥ 52 ng/L or if the absolute change to the 1-h sample was ≥ 5 ng/L. Rule-out was defined by a baseline hs-cTnT < 12 ng/L with an absolute change < 3 ng/L, or by an initial hs-cTnT below the limit of detection (LoD: < 5 ng/L) in patients presenting > 3 h after symptom onset.

In the 0/2-h protocol [11], rule-in was achieved when both baseline and 2-h hs-cTnT were ≥ 53 ng/L or when the change in hs-cTnT between samples was ≥ 10 ng/L. Rule-out required both hs-cTnT values to be < 14 ng/L with an absolute change < 4 ng/L.

For the 0/3-h protocol [12], rule-in was defined by either an initial hs-cTnT ≤ 14 ng/L followed by a rise > 7 ng/L at 3 h, or both measurements > 14 ng/L with a relative increase > 20%. Rule-out was defined as an initial hs-cTnT ≤ 14 ng/L with a second value ≤ 14 ng/L or an absolute change ≤ 7 ng/L, or an initial hs-cTnT > 14 ng/L with a second value ≤ 14 ng/L or a relative change ≤ 20%.

Detailed thresholds and timing rules are provided in Supplemental Table S1.The final diagnosis was made by the treating physician based on the third UDMI and all available clinical information [15]. For research purposes, all diagnoses were re-adjudicated by two cardiologists who were not involved in the initial management. In case of uncertainty, a third cardiologist was consulted.

### Classification of atrial fibrillation

We distinguished 4 types of AF (paroxysmal, persistent, long persistent, and permanent) as recommended by the 2020 ESC Guidelines for the diagnosis and management of AF [16]. Uncontrolled AF was defined as heart rate of 110 bpm or more. Non-valvular AF was defined as any AF that did not meet the criteria of valvular AF. Valvular AF was defined as patients with mechanical prosthetic heart valve(s) or moderate to severe mitral stenosis [17]. Left ventricular ejection fraction (LVEF) was categorized according to the guidelines recommended by the American Society of Echocardiography (ASE) and the European Association of Cardiovascular Imaging (EACVI) [18].

### Angiographic criteria for type 1 and type 2 MI

In clinical routine, further differentiation of NSTEMI into type 1 and type 2 MI was not performed routinely. For this study, infarct subtypes were adjudicated by two experienced invasive cardiologists, with a third cardiologist consulted in case of disagreement, based on systematic review of angiographic findings in conjunction with clinical presentation and biomarker dynamics at index presentation. Type 1 NSTEMI was defined per 4th UDMI definition in the presence of a spontaneous MI related to a primary coronary event. Angiographic criteria included (i) a clearly identifiable flow-limiting culprit lesion judged to be causally related to the clinical presentation and dynamic troponin rise and/or fall; (ii) angiographic evidence of plaque rupture, erosion, fissure, or dissection with or without visible intracoronary thrombus; or (iii) impaired antegrade coronary artery flow (TIMI grade < 3) in the territory of the presumed culprit lesion or periprocedural administration of a Glycoprotein IIb/IIIa inhibitor [1]. Because direct visualization of plaque rupture or thrombus is uncommon on conventional angiography in routine clinical practice, mandatory demonstration of these features was not required. In cases without visible thrombus, classification as type 1 MI required a concordant clinical presentation, dynamic biomarker profile, and the presence of a flow-limiting stenosis considered responsible for the ischemic event by both adjudicating cardiologists. Revascularization status alone was not sufficient to define infarct subtype, and cases were adjudicated independently of the final treatment strategy.

Type 2 NSTEMI was diagnosed in the absence of angiographic criteria for type 1 MI, with or without the presence of a relevant coronary stenosis together with ancillary criteria suggesting a mismatch between oxygen demand and supply. These ancillary criteria included the following:

- Uncontrolled AF: heart rate (HR) > 110 bpm
- Bradycardia: HR < 50 bpm
- Hypertension: systolic blood pressure (SBP) > 160 mmHg or pulmonary edema
- Hypotension: SBP < 90 mmHg or shock (no anemia)
- Anemia: hemoglobin (Hb) ≤ 9 g/dL (men), ≤ 8 g/dL (women)
- Respiratory Distress: respiratory rate (RR) > 20/min or SpO₂ < 95%
- Severe systemic infection or septic shock with hemodynamic instability
- Thyreotoxicosis

If multiple triggers were present, the most plausible and relevant factor was recorded.

Acute myocardial injury was diagnosed in the presence of elevated hs-cTnT > 99th percentile ULN with relevant rise and/or fall on subsequent measurements but in the absence of clinical criteria for NSTEMI other than palpitations or unspecific chest discomfort reported during AF.

### Endpoint definitions and follow-up

The primary goal was to determine the angiographic prevalence of type 1 and type 2 NSTEMI. In addition, exploratory clinical endpoints were assessed including composite and individual components of all-cause mortality, MI, or stroke and major bleedings according to International Society on Thrombosis and Hemostasis (ISTH) bleeding criteria [19]. We refrained from assessing cardiac mortality due to issues related to the unequivocal adjudication of death in a retrospective study. A sequential follow-up strategy minimized loss to follow-up [10]. To differentiate between ischemic and bleeding risks, strokes were defined excluding hemorrhagic stroke, while hemorrhagic strokes were categorized as ISTH major bleeding events [10].

### Statistical analysis

Continuous variables are reported as mean ± standard deviation (SD) or median with interquartile range (IQR) according to distribution; categorical variables as counts and percentages. Between-group comparisons used Student’s *t*-test or Wilcoxon rank-sum test for continuous variables, and *χ*^2^ or Fisher’s exact test for categorical variables. Time-to-event analyses used Kaplan–Meier estimates with log-rank tests. Multivariable Cox proportional hazards models, stratified by Killip class when indicated, evaluated associations with the composite endpoint; proportional hazards assumptions were assessed via Schoenfeld residuals. Variables for inclusion were selected based on clinical relevance and evidence from prior studies, with collinearity assessed and only one of each correlated pair retained in the final model. Interaction testing was performed via Wald tests on product terms. A two-sided *p* < 0.05 denoted statistical significance. Analyses were conducted in R 4.3.0 (R Foundation for Statistical Computing, Vienna, Austria).

## Results

### Baseline characteristics

**Fig. 1:**
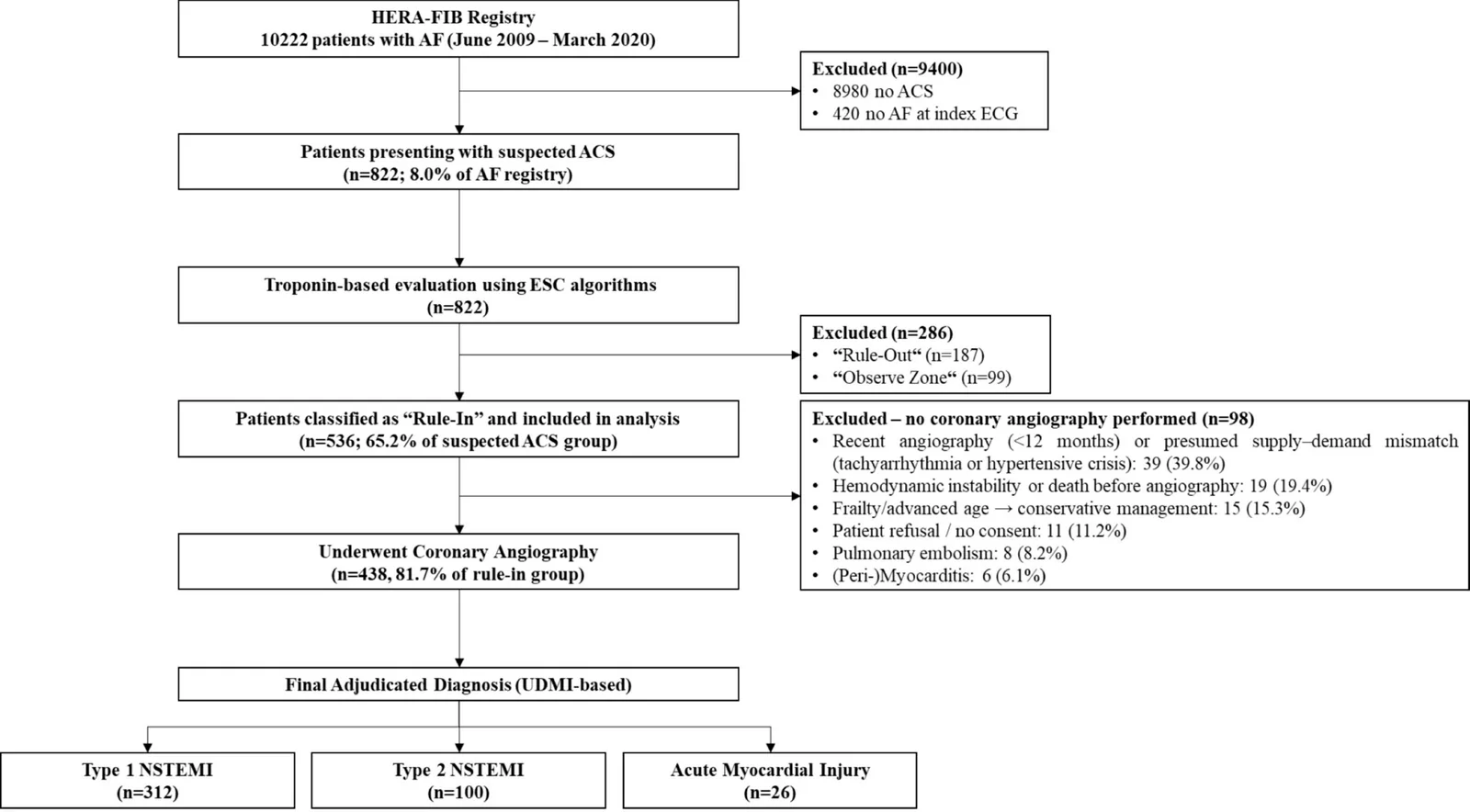
Consort diagram showing included and excluded patients in this HERA-FIB sub study. AF, atrial fibrillation; CPU, chest-pain unit; ESC, European Society of Cardiology; NSTEMI, Non–ST-elevation myocardial infarction

**Fig. 2:**
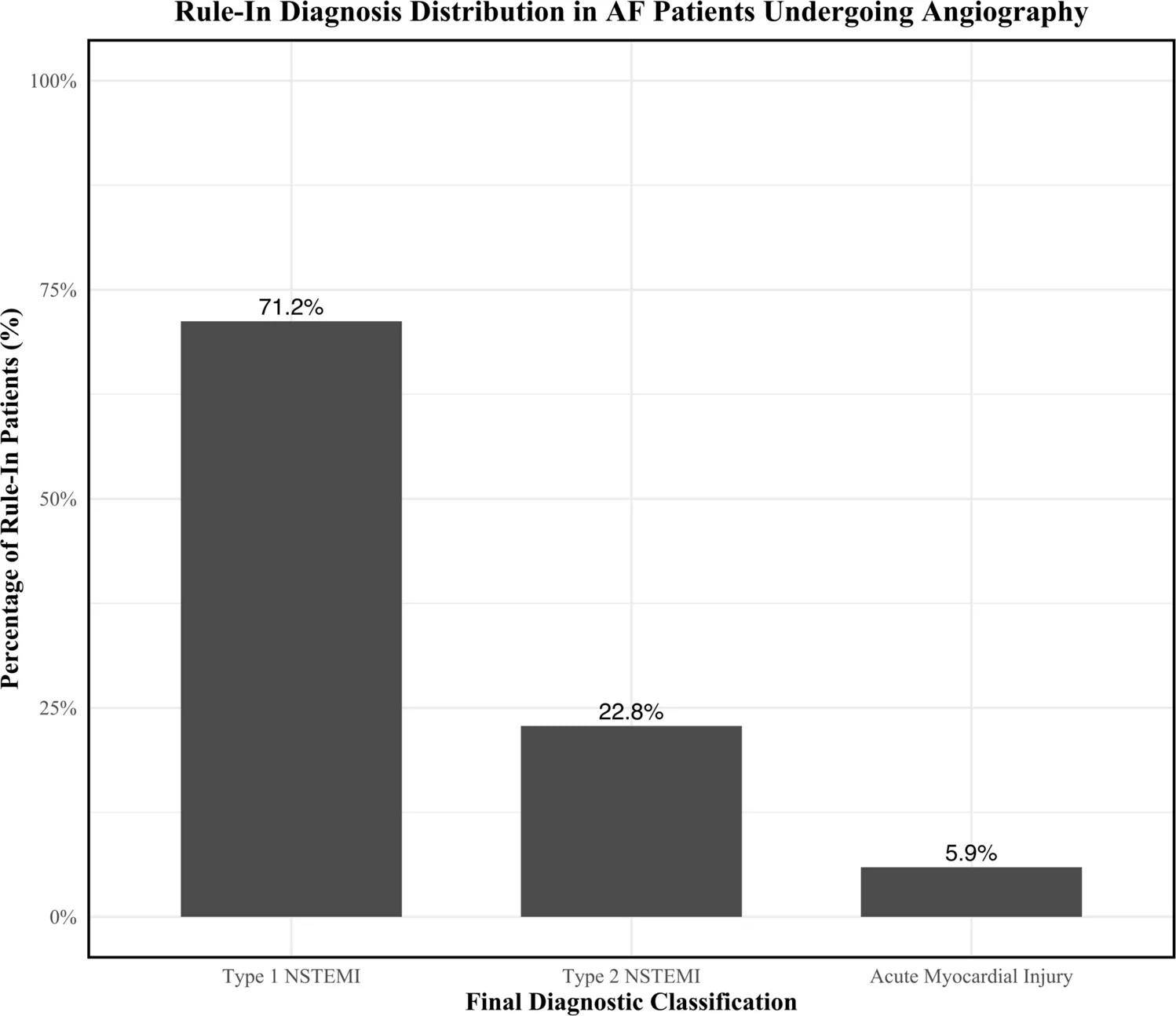
Distribution of final diagnoses in ESC rule-in AF patients undergoing coronary angiography. Among 438 symptomatic atrial fibrillation patients evaluated for suspected acute coronary syndrome and meeting ESC rule-in criteria, 312 (71.2%) were adjudicated as type 1 NSTEMI, 100 (22.8%) as type 2 NSTEMI, and 26 (5.9%) as acute myocardial injury. NSTEMI, Non–ST-elevation myocardial infarction

A total of 536 patients presented with symptomatic AF and fulfilled NSTEMI criteria based on hs-cTnT dynamics and clinical symptoms. Of these, 438 (81.7%) underwent CA during index hospitalization and formed the final study cohort. A flow diagram illustrating the composition of the final study cohort is presented in Fig. 1. Baseline characteristics stratified by NSTEMI phenotype are summarized in Table 1. Among 438 symptomatic patients evaluated for suspected ACS, 312 (71.2%) were adjudicated as type 1 NSTEMI, and 100 (22.8%) as type 2 NSTEMI (Fig. 2). A diagnosis of acute myocardial injury was made in 26 cases (5.9%) and related to pulmonary embolism (*n* = 1), myocarditis (*n* = 3), infection or inflammation including pneumonia, COPD exacerbation, or sepsis (*n* = 7), cardiogenic shock (*n* = 2), cardiac amyloidosis (*n* = 1), Takotsubo cardiomyopathy (*n* = 2), and valvular disease (severe aortic stenosis, *n* = 9; severe mitral regurgitation, *n* = 1).

**Table 1:** Baseline clinical, laboratory, echocardiographic, and angiographic characteristics by classification (type 1 NSTEMI, type 2 NSTEMI, and acute myocardial injury)

Variables	Type 1 NSTEMI (*n* = 312)	Type 2 NSTEMI (*n* = 100)	Acute myocardial injury (*n* = 26)	*p*-value
Age, y, mean (SD)	76.88 (± 8.96)	75.92 (± 9.15)	80.19 (± 9.75)	0.101
Sex, male, *n* (all)	212 (67.9)	64 (64.0)	12 (46.2)	0.073
BMI, kg/m^2^, mean (SD)	27.83 (± 4.40)	27.69 (± 5.31)	26.21 (± 4.69)	0.382
HR at admission, bpm, mean (SD)	96.11 (± 27.23)	94.30 (± 31.56)	102.23 (± 31.80)	0.451
Bpsys, mean (SD)	144.77 (± 23.95)	145.35 (± 22.88)	128.54 (± 30.95)	< 0.005
Bpdia, mean (SD)	86.84 (± 18.23)	84.93 (± 17.36)	76.54 (± 16.38)	< 0.05
**CV history**				
Arterial hypertension, *n* (all)	281 (90.4)	97 (97.0)	23 (88.5)	0.090
Diabetes mellitus, *n* (all)	101 (32.4)	29 (29)	9 (34.6)	< 0.05
Hypercholesterolemia, *n* (all)	197 (64.8)	68 (68.7)	16 (61.5)	< 0.0001
Active or ex-smoker, *n* (all)	90 (28.8)	25 (25.0)	4 (15.4)	< 0.05
CAD, *n* (all)	157 (50.3)	63 (63.0)	11 (42.3)	< 0.05
CABG, *n* (all)	48 (15.4)	20 (20.0)	4 (15.4)	0.550
MI, *n* (all)	80 (25.6)	37 (37.0)	8 (30.8)	0.088
COPD, *n* (all)	41 (13.1)	11 (11.0)	4 (15.4)	0.787
**Risk scores**				
GRACE 2.0 -score, median (IQR)	138 (124–152)	122 (106–139.2)	159.5 (137–176.8)	< 0.0001
CHA_2_DS_2_VASc-score, median (IQR)	5 (4–6)	5 (4–6)	5 (4–6)	0.001
HAS-BLED-score, median (IQR)	3 (2.8–3)	3 (2–3)	3 (2–3)	< 0.05
ORBIT-score, median (IQR)	2 (2–4)	3 (1–4)	3.5 (2–4.8)	0.0253
**Laboratory**				
hs-cTnT, ng/L, median (IQR)	129 (53.8–497)	94.5 (40–170)	250.5 (114.8–475)	< 0.005
Hb, g/dl, median (IQR)	13.2 (11.9–14.4)	13.2 (11.5–14.5)	12.1 (10.3–13.5)	< 0.05
Serum creatinine, mg/dl, median (IQR)	1.1 (0.8–1.4)	1.2 (1–1.7)	1.2 (0.9–1.9)	< 0.05
eGFR, mL/min, median (IQR)	60.8 (44.3–81.9)	56.3 (37.3–72.8)	48.4 (27–68.2)	< 0.05
CRP, mg/L, median (IQR)	8.8 (2.7–30.4)	6.5 (2–20.4)	22 (5.7–48.4)	< 0.05
NT-proBNP, ng/L, median (IQR)	4234 (2079–13,716) *n* = 111	4018 (1617.8–10,688.5) *n* = 40	10,877 (5769–18,958) *n* = 17	< 0.05
**Medication**				
Phenprocoumon, *n* (all)	143 (45.8)	44 (44.0)	14 (53.8)	0.668
DOAC, *n* (all)	83 (26.6)	27 (27.0)	2 (7.7)	0.098
LMWH, *n* (all)	40 (12.8)	15 (15.0)	3 (11.5)	0.826
ASS, *n* (all)	280 (89.7)	54 (54.0)	14 (53.8)	< 0.001
Clopidogrel, *n* (all)	211 (67.6)	22 (22.0)	8 (30.8)	< 0.001
Ticagrelor, *n* (all)	31 (9.9)	5 (5.0)	0 (0.0)	0.0089
Prasugrel, *n* (all)	8 (2.6)	0 (0.0)	1 (3.8)	0.233
Digitalis glycosides, *n* (all)	55 (17.6)	21 (21.0)	4 (15.4)	0.694
ACE inhibitors, *n* (all)	169 (54.2)	64 (64.0)	13 (50.0)	0.183
ARBs, *n* (all)	88 (28.2)	21 (21.0)	1 (3.8)	< 0.05
BB, *n* (all)	275 (88.1)	90 (90.0)	17 (65.4)	< 0.005
Diuretics, *n* (all)	109 (34.9)	41 (41.0)	14 (53.8)	0.113
Statins, *n* (all)	256 (82.1)	81 (81.0)	17 (65.4)	0.001
MRA, *n* (all)	21 (6.7)	11 (11.0)	3 (11.5)	0.309
CCB, *n* (all)	87 (27.9)	28 (28.0)	7 (26.9)	0.994
**Diagnostic work-up**				
First diagnosis of AF, *n* (all)	109 (34.9)	31 (31)	10 (38.5)	< 0.05
LVEF (*n* = 355)				
Normal, *n* (all)	47 (17.1)	27 (30.3)	4 (17.4)	0.0306
Mildly abnormal, *n* (all)	84 (26.9)	23 (23)	3 (11.5)	0.0306
Moderate abnormal, *n* (all)	77 (24.7)	22 (22)	8 (30.8)	0.0809
Severely abnormal, *n* (all)	67 (21.5)	17 (17)	8 (30.8)	0.0150
CAD				
0VD, *n* (all)	0 (0)	16 (16.0)	8 (30.8)	< 0.001
1VD, *n* (all)	37 (11.9)	13 (13.0)	2 (7.7)	< 0.001
2VD, *n* (all)	52 (16.7)	18 (18.0)	5 (19.2)	< 0.001
3VD, *n* (all)	223 (71.5)	53 (53.0)	11 (42.3)	< 0.001

*ASS* acetylsalicylic acid, *ACE*_*i*_ angiotensin-converting enzyme inhibitors, *ARB* angiotensin receptor blockers, *BB* beta blockers, *BMI* body mass index, *Bp* blood pressure, *CA* calcium channel blockers, *CABG* coronary artery bypass graft, *CAD* coronary artery disease, *CRP* C-reactive protein C, *dia* diastolic, *DOAC* direct oral anticoagulant, *eGFR* estimated glomerular filtration rate, *HR* heart rate, *Hb* hemoglobin, *hs-cTnT* high sensitive troponin T *LMWH* low molecular weight heparin, *LVEF* left ventricular ejection fraction *MI* myocardial infarction, *MRA* mineralocorticoid receptor antagonists, *NT-proBNP*, 0VD zero-vessel disease, *1VD* one-vessel coronary artery disease, *2VD* two-vessel disease, *3VD* three-vessel disease

Revascularization was uncommon in type 2 NSTEMI patients, with 7% (7 of 100) treated with PCI, whereas 310 of 312 cases with type 1 NSTEMI (99.4%) underwent revascularization and only two (0.6%) were managed conservatively.

**Fig. 3:**
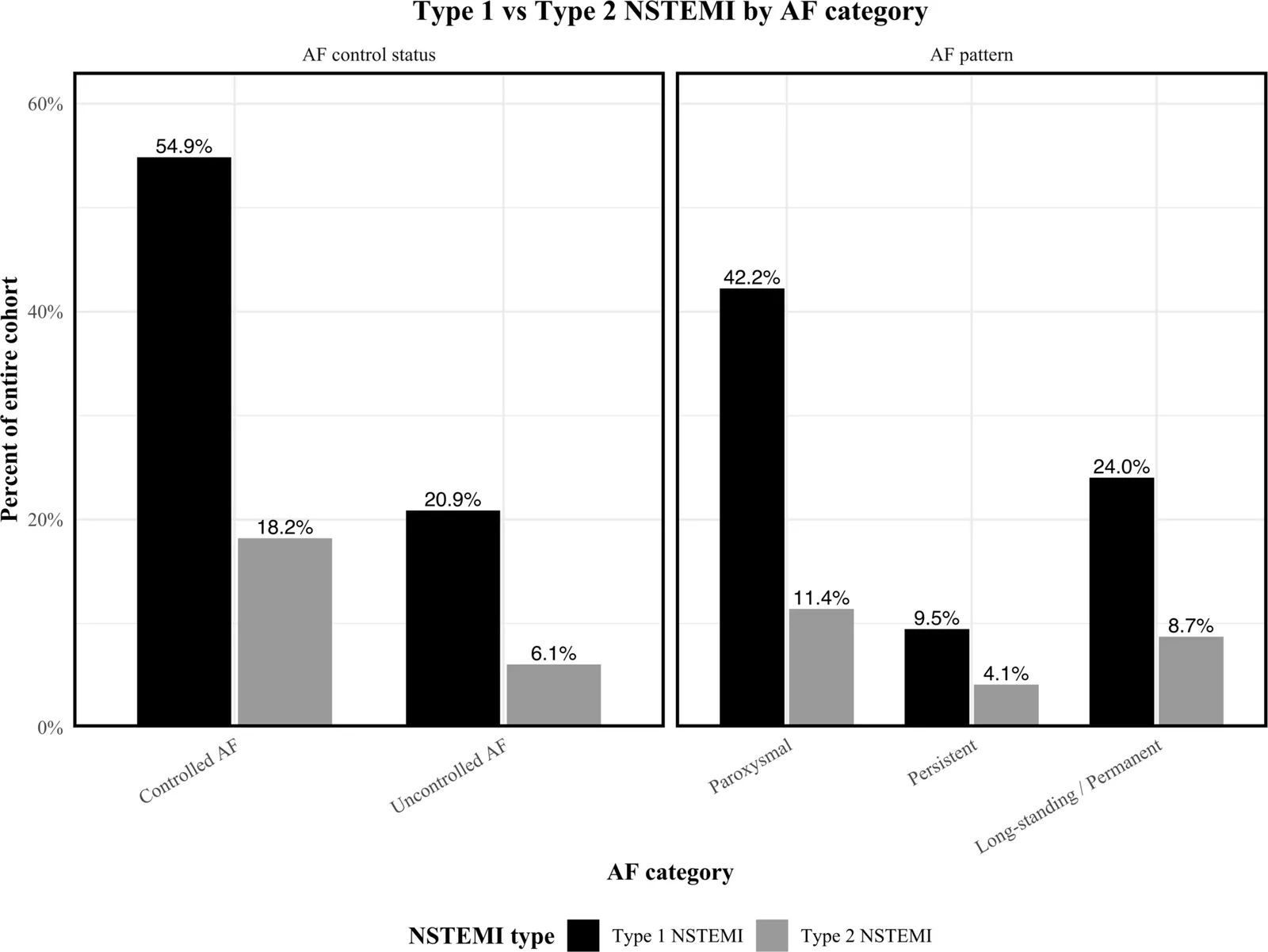
Distribution of NSTEMI subtype according to atrial fibrillation phenotype and heart rate control status. Distribution of type 1 and type 2 NSTEMI stratified by atrial fibrillation (AF) subtype (paroxysmal, persistent, and long-standing/permanent) and by heart rate control at presentation (controlled vs. uncontrolled AF). Bars represent the proportion of each MI subtype within the respective AF category. No substantial shift in subtype distribution was observed across AF phenotypes or heart rate control status. AF, atrial fibrillation; MI, myocardial infarction; NSTEMI, Non–ST-elevation myocardial infarction

Overall, the study cohort was elderly (mean age 77 [± 9.04]) and the majority was male (65.2%). Prevalence of hypertension, hypercholesterolemia, and diabetes were similar. There were no differences regarding the type of AF or the CHA₂DS₂-VASc Score between type 1 and type 2 MI. The distribution of type 1 and type 2 NSTEMI according to AF subtype (paroxysmal, persistent, and long-standing/permanent) and according to heart rate control status is shown in Fig. 3. No marked shift in subtype distribution was observed across AF categories. Detailed numerical data are provided in Supplemental Table S2. At presentation, mean heart rate was 96.1 ± 27.2 bpm in type 1 NSTEMI and 94.3 ± 31.6 bpm in type 2 NSTEMI (*p* = 0.451). Mean systolic blood pressure was 144.8 ± 24.0 mmHg and 145.4 ± 22.9 mmHg, respectively (*p* = 0.451), while diastolic blood pressure was 86.8 ± 18.2 mmHg versus 84.9 ± 17.4 mmHg (*p* < 0.05). Renal function was lowest in the acute myocardial injury group (median eGFR 48.4 mL/min [IQR 27–68.2]) compared with type 2 NSTEMI (56.3 [37.3–72.8]) and type 1 NSTEMI (60.8 [44.3–81.9]; *p* < 0.05). Inflammatory and myocardial strain markers were also highest in the acute myocardial injury group, with CRP 22 mg/L (5.7–48.4) and NT-proBNP 10,877 ng/L (5,769–18,958), respectively (both *p* < 0.05). The GRACE 2.0 score increased progressively from type 2 NSTEMI (122 [IQR 106–139]) to type 1 NSTEMI (138 [124–152]) and was highest in the acute myocardial injury group (159.5 [137–176.8]).

Regarding hs-cTnT, patients with type 1 NSTEMI exhibited significantly higher values (129 ng/L [IQR 53.8–497]) than those with type 2 NSTEMI (94.5 ng/L [IQR 40–170], *p* < 0.005). On coronary angiogram, type 1 NSTEMI was significantly more frequently associated with coronary three-vessel disease: 71.5% vs 53.0% (*p* < 0.001). In contrast, normal coronary arteries were seen in 16% of type 2 MI but no case with normal coronary arteries in patients with a type 1 MI (0% vs 16.0%; all *p* < 0.001). Furthermore, left ventricular function was more frequently impaired in type 1 NSTEMI, with normal LVEF observed in only 17.1% compared to 30.3% of type 2 cases (*p* = 0.0306). Although type 2 patients had slightly worse renal function (eGFR 56.3 vs 60.8 mL/min, *p* < 0.05), overall ischemic risk, as measured by GRACE 2.0 score, was lower than that of type 1 NSTEMI (122 vs 138, *p* < 0.0001). Pharmacological therapies differed: use of antiplatelet agents was significantly higher in type 1 NSTEMI, with aspirin administered in 89.7% versus 54.0%, clopidogrel in 67.6% versus 22.0%, and ticagrelor in 9.9% versus 5.0%. Beta-blocker use was similar in both groups (88% versus 90%).

### Secondary endpoint

**Fig. 4:**
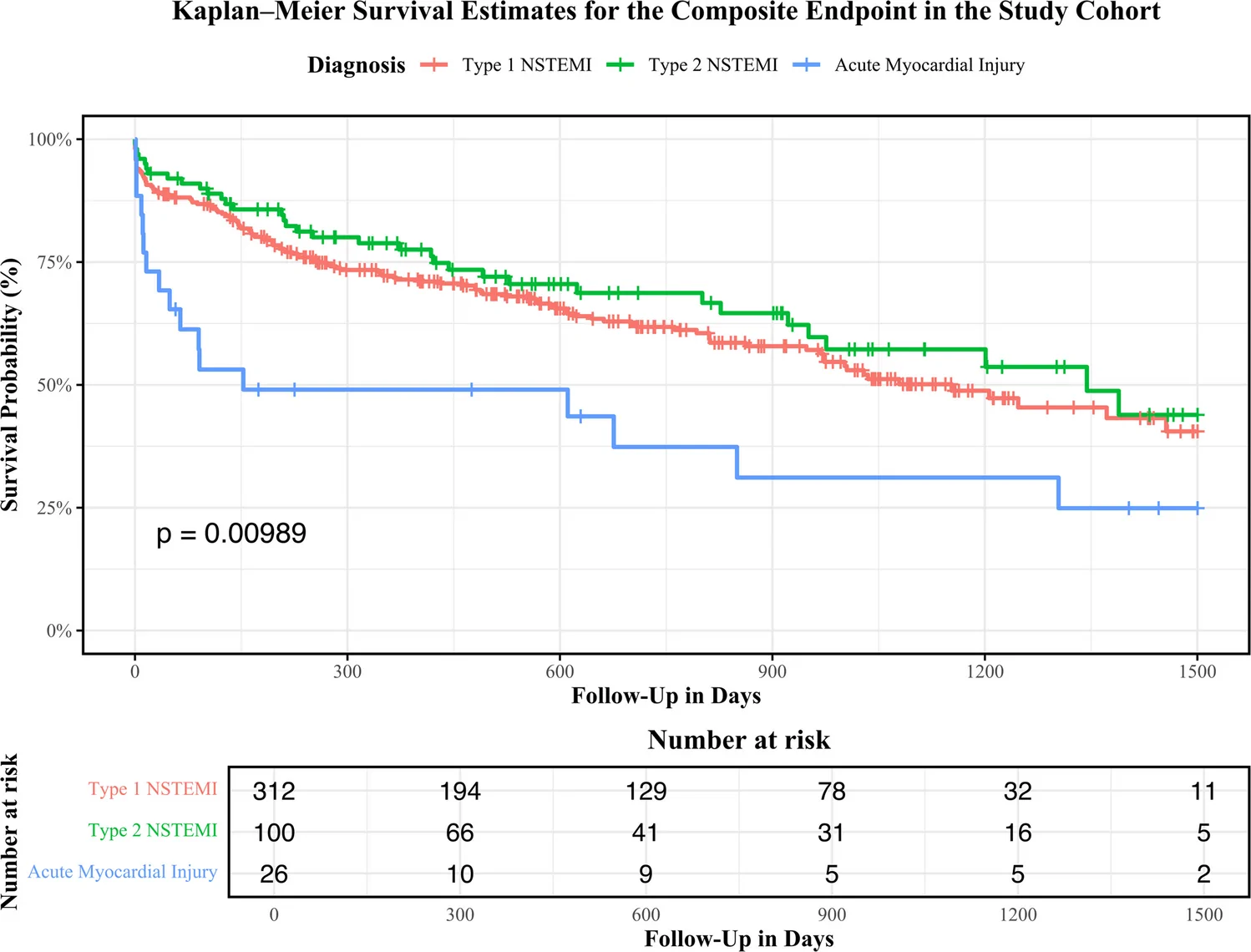
Kaplan–Meier estimates of time to composite endpoint by diagnostic category. Kaplan–Meier survival analysis showing time to the composite endpoint (all-cause mortality, myocardial infarction, stroke, or major bleeding) stratified by diagnostic category. Patients with acute myocardial injury had the poorest survival, followed by those with type 1 and type 2 NSTEMI. Differences in event-free survival were significant across groups (log-rank *p*=0.00989)

During a median follow-up of 2.21 years (IQR 1.94–2.50), the composite endpoint occurred in 40.1% of patients with type 1 NSTEMI (95% CI 34.8–45.6) and in 36.0% of those with type 2 NSTEMI (95% CI 27.3–45.8). The absolute difference in event rates between the two subtypes was + 4.1 percentage points. The event rate for all-cause mortality was higher for type 1 NSTEMI (29.8% [95% CI 25.0–35.1]) than for type 2 NSTEMI (24.0% [16.7–33.2]; HR 1.30 [95% CI: 0.83–2.00], *p* = 0.256). Major bleeding was also more frequent in type 1 (7.1% [4.7–10.4]) than in type 2 NSTEMI (3.0% [1.0–8.5]; HR 2.53 [0.76–8.46], *p* = 0.118). In contrast, recurrent MI was slightly more frequent in type 2 (12.0% [7.0–19.8]) than in type 1 NSTEMI (9.6% [6.8–13.4]; HR 0.99 [0.49–1.97], *p* = 0.971). Stroke incidence remained low and comparable between groups (3.8% vs. 4.0%; HR 1.01 [0.32–3.12], *p* = 0.991). Kaplan–Meier survival curves illustrating time to composite events across diagnostic categories are presented in Fig. 4. Across the entire cohort, the composite endpoint occurred in 40.7% after a median FU of 2.21 years.

### Predictors of the composite endpoint

After adjustment for potential confounders, coronary revascularization was independently associated with a reduced risk of the composite endpoint, with a hazard ratio (HR) of 0.89 (95% CI 0.85–0.96, *p* = 0.001). An increased risk was seen with older age (HR per year: 1.04 [1.01–1.07], *p* = < 0.05), increasing kidney failure (HR per 0.1 mg/dL: 1.04 [1.01–1.06], *p* < 0.0001), elevated NT-proBNP (HR per 1,000 ng/L: 1.10 [1.02–1.19], *p* < 0.05), and increasing GRACE 2.0 score (HR per 10-point increase: 1.55 [1.18–2.13], *p* = 0.002).

In addition, prior MI (HR 1.41 [1.12–1.87], *p*<0.05), history of hypertension (HR 1.45 [1.12–1.87], *p*=0.003), active or former smoking (HR 1.16 [1.04–1.28], *p*=0.006), and hypercholesterolemia (HR 1.16 [1.10–1.28], *p*<0.05) independently increased the risk for the composite endpoint. Details on predictors and associated HRs are provided in Table 2.

**Table 2:** Multivariate analysis for the prediction of the composite secondary endpoint (all-cause mortality, myocardial infarction, stroke, or major bleeding)

Predictor	HR (95% CI)	*p*-value
Myocardial revascularization	0.89 (0.85–0.96)	0.001
Age per 1 year (y)	1.04 (1.01–1.08)	< 0.05
Female gender	0.72 (0.44–1.17)	0.181
Serum creatinine (per 0.1 mg/dL)	1.04 (1.01–1.06)	< 0.0001
NT-proBNP (per 1000 ng/L)	1.1 (1.02–1.19)	< 0.05
GRACE 2.0-score (per 10‑point increase)	1.55 (1.18–2.13)	0.002
History of CAD	0.76 (0.44–1.32)	0.301
Prior MI	1.41 (1.12–1.87)	< 0.05
History of PAD	1.11 (0.57–2.22)	0.765
Active or ex-smoker	1.16 (1.04–1.28)	0.006
Arterial hypertension	1.45 (1.12–1.87)	0.003
Hypercholesterolemia	1.16 (1.1–1.28)	0.027
Diabetes mellitus	1.02 (0.74–1.42)	0.892

Hazard ratios (HR) with 95% confidence intervals and p-values for predictors of death, myocardial infarction, stroke, or major bleeding in the full cohort of AF patients meeting ESC rule-in criteria

*CAD* coronary artery disease, *CI* confidence interval, *HR* hazard ratio, *MI* myocardial infarction, *PAD* peripheral artery disease

### Presumed mechanism for mismatch of oxygen supply and demand in Type 2 NSTEMI

**Fig. 5:**
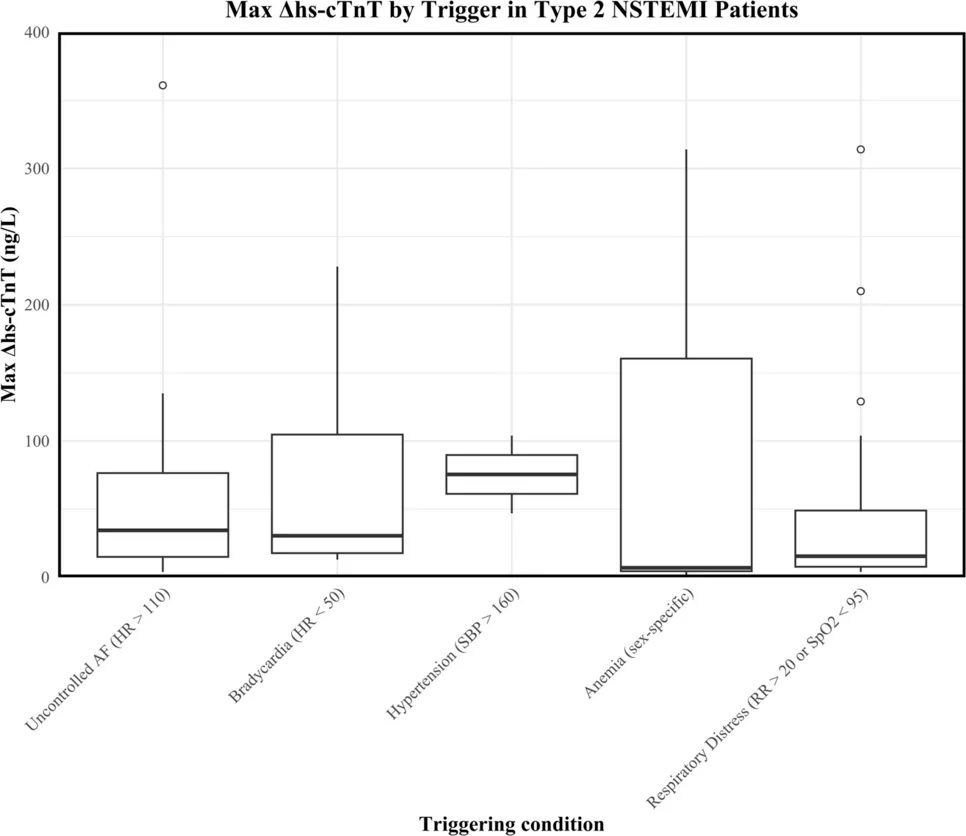
Median Δhs-cTnT by triggering condition in type 2 NSTEMI patients. Hemodynamic stressors, particularly hypertension, were associated with the largest troponin elevations, followed by rate-related triggers such as uncontrolled atrial fibrillation and bradycardia. Respiratory distress and anemia elicited smaller changes. Box plots display median values with interquartile ranges. HR, heart rate; RR, respiratory rate; SBP, systolic blood pressure; SpO2, peripheral oxygen saturation

Among patients with type 2 NSTEMI, hs-cTnT-concentration changes varied depending on the triggering condition. Hemodynamic factors elicited the largest troponin changes, with hypertension (SBP > 160 mmHg) resulting in a median concentration change of 75.5 ng/L (IQR 28.5). Tachycardias were associated with slightly smaller changes, with uncontrolled atrial fibrillation > 110 bpm associated with 34.5 ng/L (IQR 61.5). Respiratory failure was associated with a median change of 15.5 ng/L (IQR 41.2), while bradycardia was associated with a median change of 30.5 ng/L (IQR 87). Anemia led to smaller change of 7.0 ng/L (IQR 156). These variations in peak hs-cTnT change (Δhs-cTnT) across different triggers in type 2 NSTEMI patients are depicted in Fig. 5.

### Acute myocardial injury

A total of 26 patients received a diagnosis of acute myocardial injury. All patients underwent coronary angiography and additional work-up to identify the underlying etiology. Among these, ten cases had valvular heart disease (severe aortic stenosis, *n* = 9; severe mitral regurgitation, *n* = 1), infection or systemic inflammation (including pneumonia, COPD exacerbation, or sepsis; *n* = 7), myocarditis (*n* = 3), Tako-Tsubo cardiomyopathy (*n* = 2), cardiogenic shock without identifiable culprit lesion (*n* = 2), pulmonary embolism (*n* = 1), and cardiac aTTR amyloidosis (*n* = 1). This group exhibited the highest composite event rate (65.4%) and all-cause mortality (57.7%), highlighting its clinical significance.

## Discussion

In this large, real-world cohort of patients with AF and NSTEMI defined according to the 4th UDMI, of whom 81.7% underwent coronary angiography during index hospitalization, we report three major findings.

First, the majority of symptomatic AF patients presenting to the emergency department with NSTEMI were diagnosed as type 1 myocardial infarction and required coronary revascularization at index presentation, predominantly percutaneous coronary intervention (PCI). This finding is particularly noteworthy given that NSTEMI in the presence of AF is frequently assumed to be type 2 MI based on presumed oxygen supply–demand mismatch in the absence of angiographic data [5, 6]. Hence, in cohorts where classification relies primarily on clinical judgment or consensus-based adjudication without systematic angiography, misclassification between type 1 and type 2 MI cannot be excluded [7, 8].

Unlike prior studies reporting coronary angiography rates as low as 10–36% in presumed type 2 MI populations [5, 9], our cohort achieved an angiography rate of 81.7%, among the highest reported in real-world registries. Our cohort enables near-systematic angiographic assessment, thereby minimizing misclassification bias and enhancing mechanistic interpretation. This may reflect how systematic angiography enhances both the recognition of type 1 MI and the delivery of timely revascularization and tailored secondary prevention [20].

From a clinical perspective, the pre-test probability of obstructive coronary artery disease should also be considered when AF patients present with NSTEMI. Traditional atherosclerotic risk factors such as older age, diabetes mellitus, hypercholesterolemia, smoking, prior coronary artery disease or prior MI, together with ischemic symptoms, impaired left ventricular function, and a higher GRACE 2.0 risk score may support a higher-risk ischemic presentation and strengthen suspicion for type 1 MI [1, 7, 11, 21]. In our cohort, several of these characteristics were associated with adverse outcomes, supporting the concept that AF should not automatically be regarded as a sufficient explanation for troponin elevation in symptomatic patients.

Non-invasive coronary diagnostics may have a complementary role in selected patients, particularly when the clinical presentation is equivocal and immediate invasive management is not mandatory. Coronary CT angiography may help identify or exclude relevant coronary artery disease in clinically stable patients with lower or intermediate likelihood of plaque-related ACS, whereas stress imaging may be considered only after stabilization in selected cases [11, 22]. However, in patients with symptoms suggestive of ACS and dynamic troponin changes fulfilling NSTEMI criteria, our data support a low threshold for invasive coronary workup, because reliance on presumed oxygen supply–demand mismatch alone may lead to underdiagnosis of type 1 MI [11].

Importantly, this rate reflects an exceptionally high level of invasive evaluation in routine care and is in line with international benchmarks, considering that 100% adherence is rarely achievable due to unavoidable exclusions such as patient frailty, comorbidities, or advance directives.

Efforts to differentiate type 1 from type 2 MI have included a range of strategies: comparisons of baseline and dynamic cardiac troponin levels, incorporation of additional biomarkers such as fatty acid–binding protein (FABP), myoglobin, C-reactive protein (CRP), and NT-proBNP, as well as comprehensive clinical models with or without biomarkers, and more recently, omics-based classifiers [23–25]. More recently, several artificial intelligence (AI) tools have been developed and validated to discriminate type 1 from type 2 MI and hence to overcome limitations of traditional predictive models [26–28]. However, their utility remains questionable since the prevalence of type 2 MI may have been systematically overestimated and conversely the prevalence of type 1 MI underestimated as coronary angiography was not obligatory for the definition of type 2 MI in AI-based models [26, 27]. Although some AI models have demonstrated excellent agreement in validation cohorts [28], this may partly reflect the use of standardized UDMI criteria for type 2 MI, which do not require coronary angiography [1]. A second important reason that facilitates high levels of agreement is an established putative but biologically plausible pathomechanism in combination with the commonly applied methodology to reach consensus [23, 29].

Second, type 2 NSTEMI represents a clinically distinct entity that, while sharing key risk factors with type 1 NSTEMI, differs meaningfully in demographic and clinical profile. Patients with type 2 MI were slightly older and similarly distributed by sex, though neither difference was statistically significant. More notably, they had a higher prevalence of arterial hypertension (97.0% vs. 90.4%) and a greater burden of pre-existing cardiovascular disease, including more frequent prior coronary artery disease (63.0% vs. 50.3%) and myocardial infarction (37.0% vs. 25.6%).

Renal function was worse in type 2 MI, with lower median eGFR and higher serum creatinine (both *p* < 0.05), accompanied by elevated inflammatory and cardiac strain markers (CRP, NT-proBNP), suggesting greater systemic myocardial stress. Despite greater systemic stress, left ventricular function was better preserved, with normal LVEF seen in 30.3% compared to 17.1% in type 1 (*p* = 0.0306).

Anatomically, type 2 MI was associated with less extensive coronary artery disease: 16.0% had no significant lesions (vs. 0% in type 1), and only 53.0% had three-vessel disease (vs. 71.5%, *p* < 0.001). Medication use mirrored these differences, with markedly lower rates of antiplatelet therapy in type 2 MI.

Taken together, these findings reinforce that type 2 NSTEMI is not a milder variant but a distinct clinical syndrome, often driven by systemic triggers and non-obstructive coronary pathology. This distinction is crucial for guiding diagnostic workup, revascularization decisions, and individualized antithrombotic strategies. In addition, patients with type 2 MI who exhibit impaired left ventricular function or clinical heart failure should receive guideline-directed medical therapy analogous to patients with type 1 MI, including beta-blockers, renin–angiotensin system inhibition, mineralocorticoid receptor antagonists, and sodium–glucose cotransporter-2 inhibitors, as recently emphasized in expert consensus statements. Although randomized data are lacking, the myocardial injury and subsequent remodeling risk are not fundamentally different, and omission of heart failure therapy in this setting may represent a missed opportunity for secondary prevention [30].

Lastly, we identified a small but high-risk subgroup of patients classified as having acute myocardial injury. Although these individuals did not meet the clinical criteria for NSTEMI, they exhibited dynamic troponin elevations and were ultimately found to have diverse underlying etiologies, including infection, cardiomyopathy, pulmonary embolism, and valvular heart disease. Notably, this group demonstrated the highest unadjusted rates of both composite adverse events (65.4%) and all-cause mortality (57.7%), exceeding those observed in either NSTEMI subtype. Despite the absence of ischemic symptoms, nearly 70% had angiographically confirmed CAD, and 42.3% had three-vessel disease.

These findings highlight a critical diagnostic blind spot: patients with non-ischemic troponin elevations may still harbor significant coronary pathology and carry all-cause mortality rates approaching 60% during follow-up. Their heterogeneity and disproportionately poor outcomes underscore the need for more refined diagnostic tools and perhaps a re-evaluation of how this category is clinically managed. Further studies are needed to assess whether selected patients with acute myocardial injury, particularly those with underlying CAD or major systemic stressors, may benefit from invasive evaluation and tailored secondary prevention.

### Limitations

This study’s retrospective, observational design limits causal inference and renders findings vulnerable to residual confounding. Although myocardial infarction type was adjudicated using 4th UDMI criteria and considered only patients who underwent CA during index hospitalization, a misclassification between type 1 and type 2 MI cannot be fully ruled out, particularly because the spatial resolution of CA is not high enough to allow clear visualization of small erosion, fissure, or dissection of coronary plaque and intracoronary thrombi [31]. Importantly, during the recruitment period (2009–2020), intravascular imaging with optical coherence tomography (OCT) or intravascular ultrasound (IVUS) was not performed, especially in the setting of acute myocardial infarction. For this purpose, systematic evaluation with intravascular imaging including IVUS and OCT would be required in forthcoming studies.

Second, although 81.7% of eligible AF patients with NSTEMI underwent coronary angiography—a rate among the highest reported in the literature—this may still introduce a degree of selection bias. However, at the University hospital of Heidelberg, all patients who fulfill the criteria for NSTEMI are considered for coronary angiography unless there are relevant comorbidities such as severe cognitive impairment or severe dementia, advanced cancer with reduced life expectancy, concomitant severe sepsis or septic shock, or upon patients’ request. Therefore, our cohort reflects a nearly unselected, real-world population of patients undergoing early invasive evaluation. Overall rates of coronary angiography and myocardial revascularization were the highest among international PCI centers [32–35]. We also lacked data on exact symptom onset because patients frequently cannot reliably recall exact time of symptom onset. Moreover, coronary anatomy was described only semi-quantitatively, based on the presence and estimated severity of coronary stenosis based on a 50% luminal obstruction threshold, but lacked information on plaque characteristics, plaque composition, lesion complexity or functional significance of the culprit lesion. However, all coronary angiographies were re-evaluated by experienced invasive cardiologists for the presence of either visible plaque rupture, erosion, fissure or dissection, for visible intracoronary thrombus or for reduced TIMI flow.

Despite these limitations, the strength of this study lies in the large number of patients presenting with AF to an ED of a single academic center with established invasive protocols and who systematically underwent CA when the criteria of NSTEMI were present. Such a strategy provides for the first time a transparent estimation of the true rates of type 1 and type 2 MI in AF.

## Conclusion

In conclusion, this study provides a real-world characterization of NSTEMI subtypes in symptomatic AF patients undergoing systematic coronary angiography. Type 1 NSTEMI was found in 71.2% of patients undergoing angiography and therefore more frequently than reported in cohorts with lower rates of invasive evaluation. These findings suggest that limited use of coronary angiography may influence infarct subtype classification and subsequent management decisions. We also provide data on the prevalence, clinical characteristics, and outcomes of patients with acute myocardial injury, confirming that this entity is prognostically important, particularly in patients without ACS, and highlighting the need for a thorough diagnostic work-up to identify and treat the exact underlying reason. Future studies should define how clinical risk profiling and non-invasive coronary imaging can complement invasive strategies in AF patients with suspected NSTEMI.

## Supplementary Information

Below is the link to the electronic supplementary material.

Supplementary file1 (DOCX 20.0 KB)

## Data Availability

The data underlying this article will be shared on reasonable request to the corresponding author.

## Abbreviations

AF: Atrial fibrillation
CA: Coronary angiography
hs-cTnT: High-sensitivity cardiac troponin T
GRACE: Global Registry of Acute Coronary Events
ISTH: International Society on Thrombosis and Hemostasis
LVEF: Left ventricular ejection fraction
MI: Myocardial infarction
NSTEMI: Non–ST-elevation myocardial infarction
PCI: Percutaneous coronary intervention
UDMI: Universal definition of myocardial infarction
