# Correction: Type 1 and type 2 NSTEMI in atrial fibrillation: insights from the HERA-FIB registry

**DOI:** 10.1007/s00392-026-02932-4

**Published:** 2026-05-11

**Authors:** Mustafa Yildirim, Christian Salbach, Johannes Dürr, Matthias Mueller-Hennessen, Hugo A. Katus, Norbert Frey, Evangelos Giannitsis

**Affiliations:** 1 Department of Internal Medicine III, Cardiology, University Hospital of Heidelberg, 69120 Heidelberg, Germany; 2 DZHK (German Centre for Cardiovascular Research), Standort Heidelberg/Mannheim, 69120 Heidelberg, Germany

**Correction: Clinical Research in Cardiology**

https://doi.org/10.1007/s00392-026-02912-8

When this article was first published, three parts of the text were unfortunately not corrected:

1. The first sentence of the last paragraph of "Predictors of the composite endpoint" was given as

"In addition, prior MI (HR 1.62 [1.08–2.45], p < 0.05), history of hypertension (HR 1.45 [1.12–1.87], p = 0.003), active or former smoking (HR 1.16 [1.04–1.28], p = 0.006), and hypercholesterolemia (HR 1.16 [1.10–1.28], p < 0.05) independently increased the risk for the composite endpoint."

It should correctly read:

"In addition, prior MI (HR 1.41 [1.12–1.87], p < 0.05), history of hypertension (HR 1.45 [1.12–1.87], p = 0.003), active or former smoking (HR 1.16 [1.04–1.28], p = 0.006), and hypercholesterolemia (HR 1.16 [1.10–1.28], p < 0.05) independently increased the risk for the composite endpoint."

2. In the legend to Fig. 4, the last sentence was given as

"Differences in event-free survival were significant across groups (log-rank p 0.0079)"

It should correctly read:

"Differences in event-free survival were significant across groups (log-rank p = 0.00989)"

3. The first sentence of the figure legend to Fig. 5 was given as:

"Median Δhs-cTnT by triggering condition in type 2 NSTEMI patients. Hemodynamic stressors, particularly hypertension, were associated with the largest troponin elevations, followed by raterelated triggers such as uncontrolled atrial fibrillation and tachyarrhythmia. Respiratory distress, bradycardia, and anemia elicited smaller changes."

It should correctly read:

"Median Δhs-cTnT by triggering condition in type 2 NSTEMI patients. Hemodynamic stressors, particularly hypertension, were associated with the largest troponin elevations, followed by rate-related triggers such as uncontrolled atrial fibrillation and bradycardia. Respiratory distress and anemia elicited smaller changes."

The Original Article has been corrected.

