# Supplementary material for: Type 1 and type 2 NSTEMI in atrial fibrillation: insights from the HERA-FIB registry
Source: Clin Res Cardiol. 2026 Apr 15;115(10):1856–69. doi: 10.1007/s00392-026-02912-8 (PMC13562221; doi:10.1007/s00392-026-02912-8)
Supplement: Supplementary file 1 [file 392_2026_2912_MOESM1_ESM.docx]

**Supplement: Type 1 and Type 2 NSTEMI in Atrial Fibrillation: Insights From the HERA-FIB Registry**

**Supplementary Tables**

***Table S1: Information on acute coronary syndrome classification algorithms respecting sampling time intervals.***

| **Elapsed Sampling Time** | **hs-TroponinT Levels & Changes** | **Classification & Interpretation** |
| --- | --- | --- |
| **Immediate classification (ESC 0h strategy)** |  |  |
| Any time if symptoms started **>3 hours ago** | hs-TroponinT (first test) **<5 ng/L** | **Rule-out (ESC 0h)** – |
| Any time | hs-TroponinT (first test) **≥52 ng/L** | **Rule-in (ESC 0h)** |
| **30 to 90 minutes between tests (0/1h strategy)** |  |  |
|  | hs-TroponinT (first test) **<12 ng/L** AND absolute change **<3 ng/L** | **Rule-out (ESC 0/1h)** |
|  | hs-TroponinT (first test) **≥12 ng/L** OR absolute change **≥3 ng/L** but **<5 ng/L** | **Observation zone (ESC 0/1h)** |
|  | Absolute change **≥5 ng/L** | **Rule-in (ESC 0/1h)** |
| **90 to 150 minutes between tests (0/2h strategy, Reichlin 2015)** |  |  |
|  | hs-TroponinT (first test) **<14 ng/L** AND hs-TroponinT (second test) **<14 ng/L** AND absolute change **<4 ng/L** | **Rule-out (0/2h)** |
|  | Any of the following: hs-TroponinT (first or second test) **≥14 ng/L**, or absolute change **≥4 ng/L**, but hs-TroponinT values remain **<53 ng/L** and absolute change **<10 ng/L** | **Observation zone (0/2h)** |
|  | hs-TroponinT (first and second test) **≥53 ng/L** | **Rule-in (0/2h)** |
|  | Absolute change **≥10 ng/L** | **Rule-in (0/2h)** |
| **More than 150 minutes between tests (0/3h strategy)** |  |  |
|  | hs-TroponinT (first test) **≤14 ng/L** AND (hs-TroponinT (second test) **≤14 ng/L** OR absolute change **≤7 ng/L**) | **Rule-out (ESC 0/3h)** |
|  | hs-TroponinT (first test) **>14 ng/L** AND (hs-TroponinT (second test) **≤14 ng/L** OR relative change (absolute change / first test value) **≤0.2**) | **Rule-out (ESC 0/3h)** |
|  | hs-TroponinT (first test) **≤14 ng/L** AND hs-TroponinT (second test) **>14 ng/L** AND absolute change **>7 ng/L** | **Rule-in (ESC 0/3h)** |
|  | hs-TroponinT (first test) **>14 ng/L** AND hs-TroponinT (second test) **>14 ng/L** AND relative change **>0.2** | **Rule-in (ESC 0/3h)** |
|  |  |  |

***Table S2: Distribution of Type 1 vs Type 2 NSTEMI by AF categories.***

| **AF category** | **Type 1 NSTEMI** | **Type 2 NSTEMI** | **p-value** |
| --- | --- | --- | --- |
| Controlled AF | 226 (75.1%) | 75 (24.9%) | 0.6980 |
| Uncontrolled AF | 86 (77.5%) | 25 (22.5%) | 0.3718 |
| Paroxysmal | 174 (78.7%) | 47 (21.3%) | 0.3718 |
| Persistent | 39 (69.6%) | 17 (30.4%) | 0.5994 |
| Long-standing / Permanent | 99 (73.3%) | 36 (26.7%) | 0.6980 |

Abbreviations: AF, atrial fibrillation; NSTEMI, Non–ST-elevation myocardial infarction.
